# Indian psychiatry and research in Pakistan

**DOI:** 10.4103/0019-5545.69215

**Published:** 2010-01

**Authors:** Haroon Rashid Chaudhry

**Affiliations:** Department of Psychiatry, Fatima Jinnah Medical College / Sir Ganga Ram Hospital, Lahore, Pakistan

**Keywords:** Stigmatization, treatment models: treatment strategies, public awareness programs, mental health issues: psychiatric problems

## Abstract

In Asian culture, there is much stigmatization attached on having mental health problems and seeking help from a mental health expert. It is therefore, not surprising, that this stigmatization results in the refutation of the subsistence of a psychiatric problem in an individual and his family but also produces obstruction to help-seeking desires. To get a clear picture of the existence of psychiatric issues in the population, various research projects addressing psychiatric issues in children, women, and elderly are conducted both in Pakistan and India. A significant input has been taken from research conducted in India combating disaster management. In addition, public awareness programs are organized to provide information about common psychiatric disorders in children, adults, women, and the elderly.-Furthermore, psychiatric patients and their families are educated for the management of mental heath problems related to marriage, pregnancy, birth and hazards of smoking & substance abuse in young adults. Keeping in view the similarity in cultural background, treatment models, family structure, and psychosocial factors, collaborative research studies should be encouraged leading to improvement in psychiatric care of the patients both in India and Pakistan.

Mental illness has reached alarming proportions over the globe and has become a vitally important issue for the nations in terms of morbidity and mortality, as also a huge economic burden. Apart from the established biological and genetic reasons, the current disruption of the social fabric as a result of the changing political scenario, violence, and terrorism has affected the psyche of millions of individuals in this era.[1]

Pakistan has a population of 169,175,000[2] and incidence of psychiatric illnesses has increased tremendously in the recent past. It has been reported by the Pakistan Association of Mental Health that 15 million people in Pakistan are mentally disturbed and every tenth house has a psychiatric patient needing special attention.[3]

Dealing with mental health is still a huge burden in Pakistan, where one has to deal with its social, economic, and management implications. The government’s health policies and the World Health Organization (WHO) have strongly suggested the inclusion of psychiatric services at the primary care level. About 20% of the patients seen by primary care physicians have mental disorders.[4]

People in India and Pakistan share similarities in infrastructure and sociocultural background. Most of them speak the same languages of Hindi and Urdu, wear similar dresses, eat the same food, enjoy the same music and movies, and communicate in the same style, and on a similar wavelength.[5] For the last few years, significant collaborative work is being done between India and Pakistan in the field of mental health.

Under the umbrella of the Indo-Pak Punjab Psychiatric Society (IPPPS), three conferences have been organized so far. The recent work on ‘Community Psychiatry in Pakistan,’ was presented in IPPPS conferences addressing the similarities in infrastructure and sociocultural background between India and Pakistan. This study intends to incorporate a brief introduction to the mental health facilities in Pakistan and highlights community services that pay major attention to promoting mental health across the borders.[5]

As in a typical Asian culture, awareness toward psychiatric research has developed in phases in Pakistan. During the first phase, a preliminary evaluation was made of the needs and demands for mental health services in the community. This was followed by the preparation of training and teaching materials for primary healthcare personnel and educational material for the community. The next phase involved activities promoting mental health and prevention of mental illnesses. Educational administrators were sensitized to the need for incorporating mental health principles for improving the quality of education. The next stage involved the development of continuous public awareness programs for school teachers, parents, clergy, and faith healers. The Department of Psychiatry, Agha Khan Medical University, Karachi; Institute of Psychiatry, Rawalpindi; Department of Psychiatry, Armed Forces Medical College and Fountain House, Lahore are all facilitating public awareness programs addressing mental health issues.

In the avenue of treatment of schizophrenia, a recent study compared the effects of atypical antipsychotics, that is, Risperidone, Olanzapine, and Quetiapine on body weight, blood glucose, and serum prolactin levels. A significant difference was observed among three groups with regard to body weight and blood glucose, at the post-treatment level. Olanzapine-treated patients showed an increase in body weight and a rise in blood glucose level as compared to those treated with Risperidone and Quetiapine, while Risperidone-treated patients showed raised serum prolactin levels as compared to Olanzapine and Quetiapine-treated patients.[6]

Similarly, great emphasis is being made on the contribution to Community Psychiatry in Pakistan. Like other parts of the region, here also the focus is on detection, prevention, early treatment, and rehabilitation of emotional and behavioral disorders as they develop.[4] Fountain House has taken a lead as a model for Therapeutic Communities, for patients with psychosis. Fountain House Farm is a pioneering facility in psychiatric rehabilitation and in its effectiveness as a model for therapeutic communities.[7]

Religion and spirituality play a vital role in an individual’s personal and social life. They are part of a very powerful medium, and help in the healing process.[8] A recent publication has discussed the role of religion and spiritual healers in mental health.[9]

Another study highlighted the relationship of the birth of a female child with postpartum psychosis in both urban and rural cultural settings.[10] Addressing the same topic, in a recently completed study (in press), depression was positively correlated with the birth of a fourth female child in the absence of a male issue.[11]

Addressing drug-induced psychosis; an important study revealed that bhang-induced psychosis is an agitated psychosis with mania-like and paranoid symptoms. Furthermore, the study highlighted the rapid resolution of symptoms (within few days) when these patients were treated with antipsychotic drugs. In addition, the study emphasized the differential rates of cannabis psychosis between countries in the East and the West, and the culturally-bound practices of oral ingestion versus the smoking of cannabis.[12]

A study addressing bipolar affective disorder found that it is more common in persons with a positive family history.[13] In another study with regard to the treatment of bipolar disorder, a combined haloperidol and electroconvulsive therapy in manic patients has proved more effective than either one alone.[14]

In the field of child psychiatry, eminent mental health professionals have contributed worthwhile researches on topics addressing drug addiction among female children, childhood depression, behavioral problems in children with epilepsy, and emotional and behavioral problems among rural and urban school going children, which have all been published in well-cited journals.[15–20]

Depression in old age requires special attention. A recently published study assessed the frequency and severity of depression after the death of a spouse. Results indicated 77% males and 19% females had severe depression after the death of their spouses.[21] Many contributions in the psychogeriatric field are enlightening for us and have paved the way for further research in this area.[22–25]

In certain areas, a strong influence of Indian Psychiatry has been observed at the global level. An important contribution from India addressing mental health issues, has shared the personal experiences of Kar working in two major natural disasters, the Baripada Inferno and the Orissa Super Cyclone.[26] Kar emphasized the post-disaster psychological problems that survivors faced, as well as stressed the pertinent need for developing disaster management centers and strategies that could effectively tackle future catastrophic events. In 2006, Rana *et al*.’s contribution, addressing the needs and psychosocial relief of the survivors of the 2005 earthquake that struck Azad Jammu and Kashmir and parts of North Western Frontier Province of Pakistan, highlighted the urgency of having a national action plan that would accurately meet the needs of the survivors. This action plan introduced several core strategies that, at present, essentially serve as a foundation for future relief efforts in the country.[27] Both studies clearly identified that the only possible approach to efficiently deal with the effects of natural disasters in the future was to be proactive rather than reactive. Similarly, Mufti *et al*. undertook a study to measure the prevalence of psychiatric morbidity among Afghan refugees and it was concluded that most of the Afghan refugees had a diagnosis of post-traumatic stress disorder (PTSD).[28]

Another aspect of the influence that Indian Psychiatry has had at the global level, especially in the South Asian region, is the amount of research done on the significance of spirituality on the mental health of individuals. One of the prominent characteristics that exist among the countries in this part of the world is the profound association people have with their religious beliefs. Golecha pointed out that spirituality is intimately related to the mental processes, that is, the mind, and affects the psychic functions of an individual. It can further provide a holistic approach in improving the mental health of the afflicted, as well as promote mental health gains for individuals by improving family ties.[29] A similar study was also carried out by Chaudhry[8,9] focusing on the role and significance of spirituality in psychiatric treatment.

Malik’s extensive work on women and mental health has underlined the imperative need to not only create awareness regarding women’s issues, but also to develop substantial programs at the national level that can efficiently promote and address women’s issues and their mental health.[30] Niaz *et al*. have contributed many studies addressing women issues, that is, PTSD, depression, stress in women, women’s mental health in Pakistan, and other issues related to women.[31–37] One study that estimated the frequency of depressive disorder in the upper and upper-middle class urban women in Karachi and identified the psychosocial factors of depressive disorders in these women, showed that women in the upper and upper-middle class suffer from depressive disorders twice as much as males. The psychological factors associated with depression in this group are mainly related to marriage and role conflicts within the domestic sphere of life.[34]

There is a great need in the future to promote mental health in both India and Pakistan, and collaborative research studies should be encouraged, keeping in view the similar treatment models, family structure, and psychosocial factors.
